# Visceral hyperalgesia induced by forebrain-specific suppression of native Kv7/KCNQ/M-current in mice

**DOI:** 10.1186/1744-8069-7-84

**Published:** 2011-10-26

**Authors:** Yeping Bi, Hui Chen, Jun Su, Xu Cao, Xiling Bian, KeWei Wang

**Affiliations:** 1 Department of Neurobiology, Neuroscience Research Institute, Peking University Health Science Center, Beijing 100191, China; 2 Department of Molecular and Cellular Pharmacology, Peking University School of Pharmaceutical Sciences, 38 Xueyuan Road, Beijing 100191, China

**Keywords:** forebrain, retigabine, XE-991, capsaicin, acetic acid, c-Fos, somatosensory cortex

## Abstract

**Background:**

Dysfunction of brain-gut interaction is thought to underlie visceral hypersensitivity which causes unexplained abdominal pain syndromes. However, the mechanism by which alteration of brain function in the brain-gut axis influences the perception of visceral pain remains largely elusive. In this study we investigated whether altered brain activity can generate visceral hyperalgesia.

**Results:**

Using a forebrain specific αCaMKII promoter, we established a line of transgenic (Tg) mice expressing a dominant-negative pore mutant of the Kv7.2/KCNQ2 channel which suppresses native KCNQ/M-current and enhances forebrain neuronal excitability. Brain slice recording of hippocampal pyramidal neurons from these Tg mice confirmed the presence of hyperexcitable properties with increased firing. Behavioral evaluation of Tg mice exhibited increased sensitivity to visceral pain induced by intraperitoneal (i.p.) injection of either acetic acid or magnesium sulfate, and intracolon capsaicin stimulation, but not cutaneous sensation for thermal or inflammatory pain. Immunohistological staining showed increased c-Fos expression in the somatosensory SII cortex and insular cortex of Tg mice that were injected intraperitoneally with acetic acid. To mimic the effect of cortical hyperexcitability on visceral hyperalgesia, we injected KCNQ/M channel blocker XE991 into the lateral ventricle of wild type (WT) mice. Intracerebroventricular injection of XE991 resulted in increased writhes of WT mice induced by acetic acid, and this effect was reversed by co-injection of the channel opener retigabine.

**Conclusions:**

Our findings provide evidence that forebrain hyperexcitability confers visceral hyperalgesia, and suppression of central hyperexcitability by activation of KCNQ/M-channel function may provide a therapeutic potential for treatment of abdominal pain syndromes.

## Background

Visceral hypersensitivity is considered to be an important pathophysiologic mechanism for common abdominal pain symptoms in patients with functional gastrointestinal disorders (FGIDs) such as irritable bowel syndrome, non-cardiac chest pain and functional dyspepsia [1]. As visceral pain persists over time, it is thought that changes in the central nervous system (CNS) with altered neuronal processing and neural plasticity can ultimately lead to visceral hyperalgesia [2,3], indicating there is bidirectional brain-gut interaction in visceral pain [4].

The brain-gut axis is composed of ascending and descending pathways where gastrointestinal sensory information is transmitted to the brain through vagal and spinal afferent nerves, and vice versa. There is an emerging consensus that the CNS exerts a significant influence on the clinical presentation of pain symptoms. Findings from neuroimaging studies using functional Magnetic Resonance Imaging (fMRI), Positron emission tomography (PET) and single photon emission computed tomography (SPECT) have shown activation of brain regions in response to visceral pain stimulation, indicating involvement of brain function in modulation of visceral pain [4-8]. Although visceral hypersensitivity has been widely demonstrated in patients, the underlying CNS mechanism which accounts for this hypersensitivity remains unknown.

The forebrain functions to control and regulate cognitive, sensory and motor processing. It has been shown that excitability of forebrain regions such as the somatosensory cortex, the anterior cingulated cortex (ACC) and the insular cortex is critical for central sensitization in the ascending pathways of visceral pain [5,9,10]. Pharmacological studies have shown that centrally acting anticonvulsant compounds are effective in animal models of visceral pain, suggesting the involvement of neuronal hyperexcitability in generation of visceral pain hypersensitivity [11,12]. However, experimental evidence directly connecting central alteration of cortical excitability and sensitivity to visceral pain is lacking.

Native M-current, encoded by Kv7/KCNQ channels, is a subthreshold voltage-gated K^+ ^current that serves as a brake and suppresses abnormal ectopic discharges of neurons and control neuronal hyperexcitability [13-17]. Systemic activation of neuronal M-current by the Kv7/KCNQ channel opener retigabine results in attenuation of inflammatory, neuropathic and visceral pain [12,18-21], and decreases in neuronal excitability of noceiceptive neurons and C-type nerve fibers [13,14].

In this study, we hypothesized that altered forebrain function by enhanced neuronal excitability can influence visceral pain. To test this concept, we generated transgenic mice expressing a dominant-negative pore mutant of KCNQ2 that suppresses native KCNQ/M-current and enhances membrane excitability under the control of the forebrain specific promoter αCaMKII. Our findings show that forebrain hyperexcitability can cause visceral hyperalgesia, and pharmacological activation of central KCNQ/M channel function has potential to provide a therapeutic means for treatment of abdominal pain syndromes.

## Results

### Generation of forebrain expression of KCNQ2 G279S mutant mice

**Figure 1:**
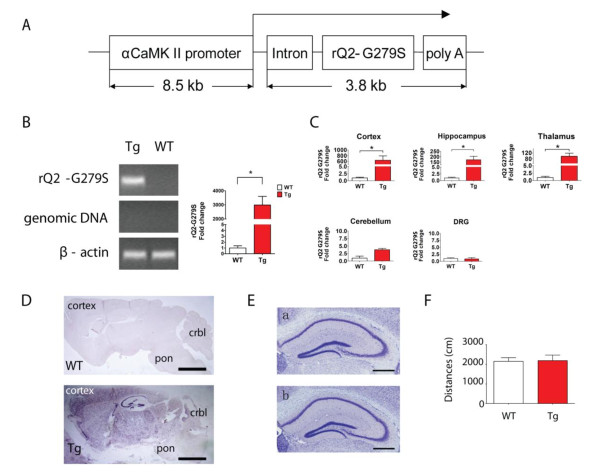
**Generation and confirmation of transgenic mice overexpressing the KCNQ2 dominant-negative pore mutant in forebrain**. A. Schematic organization of the transgene for forebrain-specific expression of the dominant-negative mutant of rat KCNQ2-G279S (rQ2-G279S) driven by the αCaMKII promoter. B. Identification of rQ2-G279S mRNA in the brain isolated from transgenic (Tg) and wild type (WT) mice by real time RT-PCR (mean ± s.e.m., n = 4, *p < 0.05). The template of rQ2-G279S specific primers for detection of KCNQ2 mutant gene expression is located in the area of SV40 poly A of the 265-plasmid (transgenic construct). The template of genomic DNA primers for detection of genomic DNA contamination is located after SV40 poly A in the 265-plasmid (transgenic construct), which does not undergo transcription. β-actin is used as an internal reference. C. Expression of rQ2-G279S mRNA isolated from 5 different tissues of WT and Tg mice by real time RT-PCR. The transgene rQ2-G279S was adequately expressed in the cortex, hippocampus and thalamus, with little expression in the cerebellum, and was not expressed in the DRG of Tg mice (mean ± s.e.m., n = 4, *p < 0.05). D. Expression of rQ2-G279S mRNA of WT (top) and Tg mice (down) using in situ hybridyzation. The transgene rQ2-G279S was adequately expressed in the cortex, hippocampus, and thalamus, with little expression in the cerebellum (crbl) and pons, and was not expressed in the medulla of Tg mice. The scale bar is 2mm. E. Nissl-stained coronal sections of hippocampus. No obvious structural change was found in adult transgenic mice (a) or wild type littermates (b). Scale bar, 500 μm. F. Locomotor activity test, no significant difference was detected in wild type (WT) and transgenic mice (Tg) in the total distance traveled during a period of 30 minutes (mean ± s.e.m., n = 7).

To investigate the effect of altered forebrain excitability on visceral pain, we generated a line of transgenic (Tg) mice that express a dominant-negative pore mutation of the rat Kv7.2/KCNQ2 channel (rQ2-G279S) in which a glycine residue (Gly, G) in the selectivity filter was mutated to Serine (Ser, S) under the control of the forebrain specific αCaMKII promoter (Figure 1A). To confirm the dominant-negative effect of mutant rQ2-G279S on wild type (WT) channel function, we tested the ability of the mutant to attenuate the KCNQ2 current upon co-expression in *Xenopus *oocytes before the generation of transgenic mice. Consistent with a previous report [22], co-expression of WT KCNQ2 with the rQ2-G279S mutant resulted in significant inhibition of the channel function (data not shown). Real-time RT-PCR analysis of whole brain mRNA showed expression only of rQ2-G279S mRNA in the Tg mice without contamination of transgenic genomic DNA bands (Figure 1B). The transgenic rQ2-G279S mRNA was robustly distributed in forebrain regions such as the cortex, hippocampus, and thalamus, but there was little or no expression in the cerebellum and dorsal root ganglion (DRG) in Tg mice (Figure 1C). *In situ *hybridization further confirmed that the transgenic rQ2-G279S mRNA was highly expressed in the forebrain (Figure 1D). These results demonstrate that the expression of dominant-negative pore mutant (rQ2-G279S) mRNA was achieved in the forebrain of Tg mice.

To determine whether any developmental defect was present, we evaluated the histology of the brain in the Tg mice. These Tg mice showed normal development of the adult hippocampus without obvious structural changes (Figure 1E). Moreover, these Tg mice did not develop any obvious difference in locomotor activity behavior (Figure 1F) or body weight, as compared with WT mice (WT 19.05 ± 0.3980g vs Tg 19.29 ± 0.4783g at the age of 8 weeks, mean ± s.e.m., n = 8-11).

### Neuronal hyperexcitability of cortex in Tg mice

**Figure 2:**
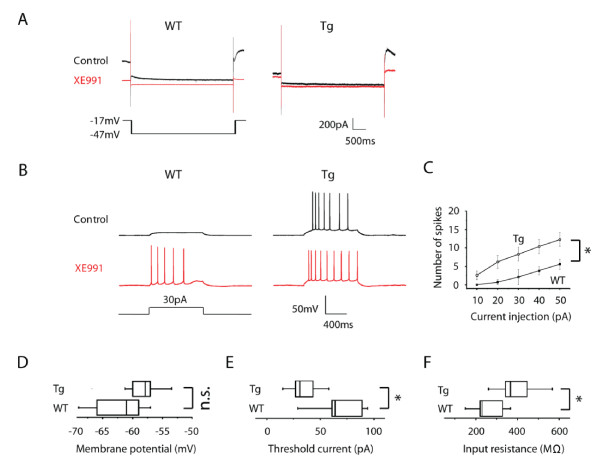
**Electrophysiological characterization of CA1 pyramidal neurons from wild type and transgenic mice**. A. Representative deactivation currents recorded from CA1 pyramidal neurons in fresh slice preparations from wild type (WT, left panel) or transgenic mice (Tg, right panel). Overlay of currents evoked by the protocol in the absence (black) or presence (red) of 10uM XE991. B. Response of CA1 pyramidal neurons with injection of 1000ms, 30pA depolarizing current pulse into the cell from WT (left panels) and Tg mice (right panels) before (black) and after (red) application of XE991. XE991 increased action potential firing in WT mice, but it was less effective in neurons from Tg mice. C. Comparison of average number of spikes evoked by injected current in WT and Tg neurons. D-F. Box plots illustrating the differences in WT and Tg mice in resting membrane potential, threshold current required for action potential firing and input resistance, n = 10; Data are shown as mean ± s.e.m., *p < 0.05.

To characterize neuronal excitability of Tg mice, we analyzed the electrophysiological properties of CA1 pyramidal neurons by brain slice recordings. In neurons from WT mice (Figure 2A, left panel), native KCNQ/M-current was recorded with the characteristic of time-dependent deactivating kinetics, which was blocked by the channel blocker XE991 (10 μM). In contrast, the neuronal M-current in Tg mice was much smaller than that of WT mice (Figure 2A, right panel). Current clamp recording of action potentials showed that WT neurons fired either no or several spikes in the presence of 10 μM XE991, upon current injection of 30 pA for 1000 ms (Figure 2B, left panels). In contrast, neurons from Tg mice fired repetitive action potentials under the same conditions (Figure 2B, right panels). The total number of spikes was significantly increased in neurons from Tg mice, as compared with WT mice (Figure 2C). The resting membrane potential of Tg mice neurons showed a shift of about 5 mV towards depolarization, as compared with WT neurons (Figure 2D). In addition, the spike firing threshold of Tg mouse neurons was lower (Figure 2E), and the input resistance in Tg mice was greater, as compared with WT controls (Figure 2F). These results confirmed the neuronal hyperexcitability of hippocampal neurons from Tg mice.

### Visceral hyperalgesia in Tg mice induced by acetic acid or magnesium sulfate

**Figure 3:**
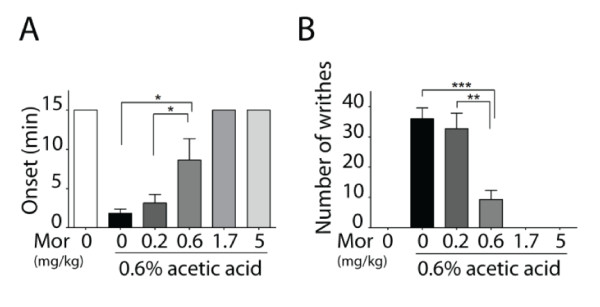
**Validation of visceral pain induced with acetic acid**. Visceral pain was induced with acetic acid in ICR species of mice. The onset (A) and the total number of writhes (B) during a period of 15 minutes were determined after s.c. administration of morphine (0.2, 0.6, 1.7, 5 mg/kg) 20 minutes before i.p. injection of 0.6% acetic acid. Intraperitoneal injection of 0.6% AA resulted in a significant reduction in onset time of writhes and increased number of writhes. Morphine significantly increased the onset and decreased the total number of writhes at a dose of 0.6-5 mg/kg (mean ± s.e.m., n = 6-10, *p < 0.05, **p < 0.01, ***p < 0.001).

**Figure 4:**
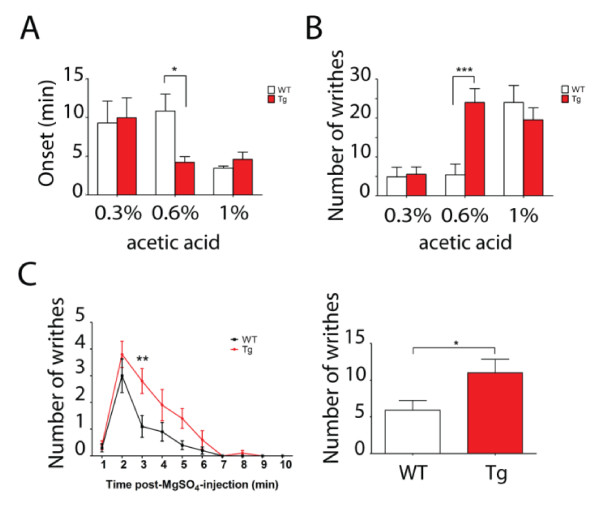
**Visceral hyperalgesia in transgenic mice induced by visceral stimulation of acetic acid or magnesium sulfate**. A-B. The onset (A) and the total number of writhes (B) during a period of 15 minutes were determined after i.p. injection of 0.3%, 0.6%, 1% acetic acid (n = 7-10). 0.6% acetic acid resulted in a shorted onset and increased number of writhes in transgenic (Tg) mice. C. Induction of visceral pain induced by magnesium sulfate (12 mg/k g, i.p.). Tg mice displayed an increase of writhes in response to magnesium sulfate during a period of 10 minutes (mean ± s.e.m., n = 10, *p < 0.05, **p < 0.01, ***p < 0.001).

Previous results showed there was increased excitability of cortical neurons from Tg mice. To assess the effect of neuronal hyperexcitability on visceral hyperalgesia, we utilized a model of visceral pain induced by acetic acid (AA). Intraperitoneal (i.p.) injection of 0.6% AA resulted in a significant reduction in the onset time of writhing and caused increased numbers of writhes, as compared with the saline control group (Figure 3). To validate this model, we tested the effect of morphine on visceral pain. Injection of different concentrations of morphine (0.6, 1.7 and 5 mg/kg, s.c.) significantly increased the onset time of writhing and decreased the total number of writhes (Figure 3), confirming that visceral pain was successfully induced by 0.6% AA. To examine the visceral pain sensitivity of Tg mice, we injected (i.p.) three different concentrations of AA (0.3%, 0.6% and 1%) and tested their effects on writhing. As shown in Figure 4A and 4B, Tg mice exhibited a significant reduction in onset time and an increased number of writhes in response to visceral stimulation by 0.6% AA during a period of 15 minutes, as compared with WT mice. These results indicate that Tg mice with forebrain hyperexcitability are more susceptible to visceral pain induced by AA.

To further confirm the result obtained from the AA test, we used another assay of visceral pain induced by magnesium sulfate. Injection (i.p.) of magnesium sulfate (12mg/kg) into WT mice resulted in the onset of writhing which lasted for several minutes before returning to the baseline level (Figure 4C, left panel). As compared with the WT group, Tg mice exhibited a significant increase in the number of writhes in response to magnesium sulfate stimulus within a period of 10 min (Figure 4C, right panel), which is consistent with the result obtained from 0.6% AA, further confirming that the Tg mice are more sensitive to visceral pain induced by chemical stimuli.

### Visceral Hyperalgesia in Tg mice induced by intracolon injection of capsaicin

**Figure 5:**
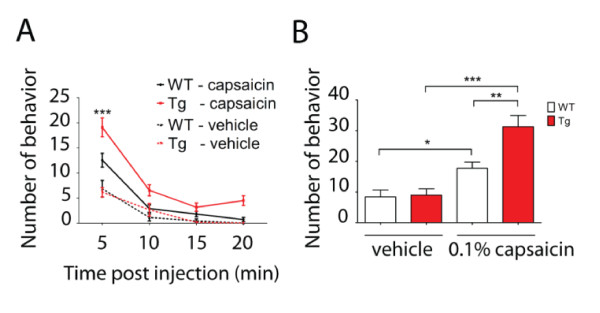
**Visceral hyperalgesia in transgenic mice induced by intracolon injection of capsaicin**. Behavioral reactions (abdominal licking, stretching, squashing the lower abdomen against floor and abdominal retractions) evoked by intracolonic injection of vehicle solution or 0.1% capsaicin in WT and Tg mice. A. Number of behaviors observed in each 5 min period over a total observation time of 20 min post-administration. ***Indicates the other three groups which were significantly different from capsaicin-treated Tg mice during the first 5 min observation period (*p *< 0.001). B. Number of behaviors observed in the total 20 min period post-administration. Data are shown as mean ± s.e.m. (n = 5-11, *p < 0.05, **p < 0.01, ***p < 0.001).

To further investigate whether altered excitability in the forebrain affects the pain sensation of abdominal viscera where most nerve endings are embedded deep within the wall of the organ, an intraluminal injection of capsaicin into the colon was performed to induce visceral pain. Intracolon administration of vehicle solution into either WT mice or Tg mice induced similar behavioral responses with a small amount of abdominal licking, which was clearly separate from normal grooming activity (Figure 5). Intracolon administration of 0.1% capsaicin into Tg mice evoked significant behavioral responses with abdominal licking, stretching, squashing of the lower abdomen against floor and abdominal retractions, as compared with the group of WT mice (Figure 5). These results indicate that Tg mice are more sensitive to visceral pain induced by intracolon chemical stimulation.

**Figure 6:**
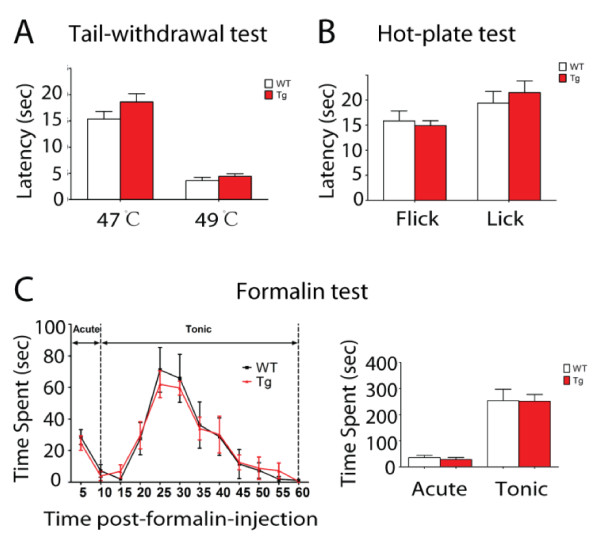
**Normal cutaneous pain sensitivity in transgenic mice to thermal and inflammatory stimulation**. A-B. Comparison of thermal sensitivity in wild type (WT) and transgenic mice (Tg) by cutaneous application of thermal stimulation. No significant difference was detected between the two groups in tail withdrawal test (A) at 47°C and 49°C and in hot plate test (B) at 53°C (mean ± s.e.m, n = 7-11). C. The Tg mice displayed equivalent licking/biting behavior in both the acute and tonic phases in 5% formalin test (mean ± s.e.m, n = 7-9). The acute phase of the formalin test was defined as 0-10 min after injection and the tonic phase as 10-60 min after injection.

To investigate whether cutaneous nociceptive behaviors of Tg mice are also affected, we utilized two models of thermal and inflamatory pain. In tail-withdrawal test, both WT and Tg mice showed equal latencies to withdraw tail from thermal stimulation with water temperature of 47°C or 49°C (Figure 6A). In another thermal test of hot-plate, both WT and Tg mice also exhibited an equal latency in response to nociceptive stimulation applied to hindpaws on a hot plate set at 53°C (Figure 6B). In formalin test, Tg mice showed similar pain responses for the time spent on licking hindpaw injected with 5% formalin in both acute and tonic phase (Figure 6C). These results suggest that both WT and Tg mice showed normal pain responses to cutaneous stimulation by heat and inflammation.

### Enhanced expression of c-Fos in the forebrain cortex of Tg mice

**Figure 7:**
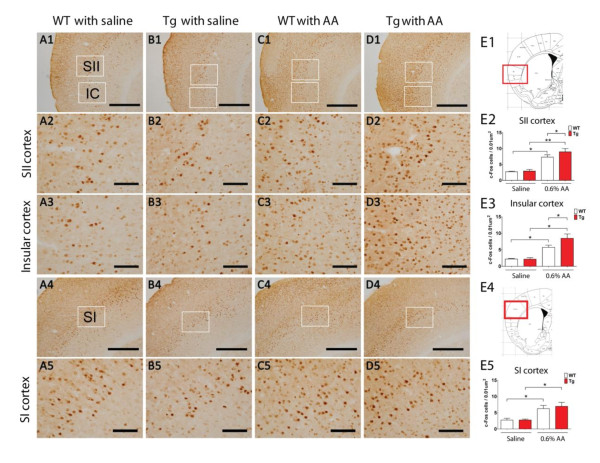
**Induction of c-fos expression in forebrain cortex by 0.6% acetic acid stimulation**. A-D. Representative photomicrographs of saline or acetic acid (i.p.) induced c-Fos expression in somatosensory SII cortex, insular cortex (IC) and SI cortex in wild type (WT) and transgenic (Tg) mice. The scale bar for panels A1-D1 and A4-D4 is 500 μm (low magnification), and the scale bar for A2-D2, A3-D3 and A5-D5 is 100 μm (high magnification). E1, Brain map [52] with photographed somatosensory SII and insular cortex framed in red. E2-3, Density of c-Fos positive cells in somatosensory SII cortex (E2) and Insular cortex (E3) of WT and Tg mice treated with saline or 0.6% acetic acid (i.p.). E4, Brain map [52] with the SI cortex framed in red. E5, Density of c-Fos positive cells in the SI cortex from WT and Tg mice treated with saline or 0.6% acetic acid (i.p.).Data are presented as mean ± s.e.m (n = 3-4, *p < 0.05, **p < 0.01).

Previous data indicate that visceral hyperalgesia is mediated by enhanced forebrain activity of Tg mice. To further confirm cortical activity is enhanced in response to visceral pain, we examined c-Fos expression in the forebrain cortex using immunohistochemistry. As shown in Figure 7, the number of c-Fos-positive cells in the somatosensory SI, SII cortex and insular cortex was increased in both WT and Tg mice after induction of visceral pain by 0.6% AA, as compared with control saline groups. In particular, the increased number of c-Fos-positive cells in the Tg group was even higher than that of the WT mice in the somatosensory SII cortex and insular cortex, but not the SI cortex (Figure 7). These results indicate that neuronal activity of the somatosensory SII cortex and insular cortex in the forebrain region of Tg mice is enhanced in response to visceral stimulation by chemical AA.

### Visceral hyperalgesia induced by intracerebroventricular injection of Kv7/KCNQ/M channel blocker

**Figure 8:**
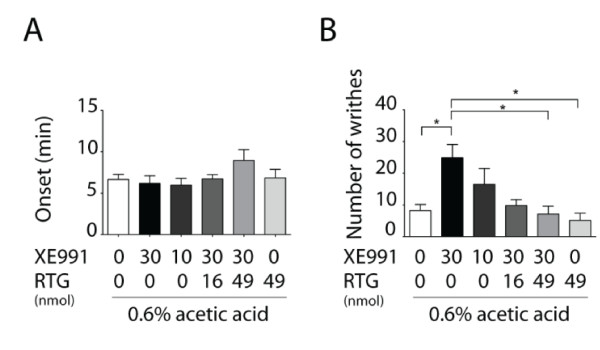
**Effect of intracerebroventricular (i.c.v.) administration of KCNQ channel modulators on visceral pain induced by acetic acid in wild type mice**. The onset (A) and the total number of writhes (B) after induction of visceral pain by 0.6% acetic acid (i.p.) were determined over a period of 15 min with XE991, retigabine (RTG) or vehicle administered by i.c.v. injection. XE991 (30 nmol) significantly increased the number of writhes, and RTG (49 nmol) reversed its effect. Data are expressed as mean ± s.e.m (n = 7-13, *p < 0.05).

To mimic the role of forebrain hyperexcitability in visceral pain, we injected Kv7/KCNQ channel modulators into the lateral ventricle of WT mice. Intraperitoneal injection of 0.6% AA into WT mice resulted in visceral pain with writhing onset time of about 6 min, which included 9 writhes during a period of 15 min. Intracerebroventricular (i.c.v.) injection of either retigabine or XE991 had no effect on writhing onset time (Figure 8A). The i.c.v. injection of the channel blocker XE991 (30 nmol) into WT mice had no effect on onset time, but significantly increased the number of writhes (about 3-fold) (Figure 8). To further confirm this effect of XE991, we co-injected (i.c.v.) XE991 with channel opener retigabine into WT mice, and tested the central effect of retigabine on visceral pain. The i.c.v. co-injection of retigabine (49 nmol) with XE991 (30 nmol) can reverse the effect of XE991 (30 nmol), leading to a significant reduction in writhes (Figure 8B). Together, these results show that i.c.v. injection of blocker XE991 can mimic visceral hyperalgesia of Tg mice, indicating that pharmacological suppression of central Kv7/KCNQ channel function can enhance visceral pain, which is consistent with the previous results obtained from Tg mice. This result also suggests that central activation of Kv7/KCNQ channel function can attenuate visceral pain.

### Attenuation of visceral pain by i.p. injection of the Kv7/KCNQ channel opener retigabine

**Figure 9:**
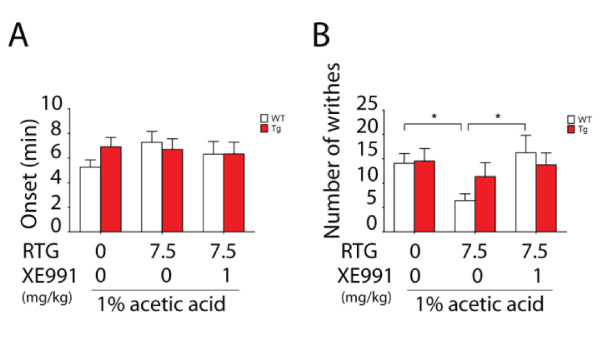
**Antinociceptive effects of KCNQ channel opener retigabine on visceral pain induced by acetic acid in wild type mice, but not transgenic mice**. The onset time (A) and the total number of writhes (B) were determined with vehicle or retigabine (RTG, 7.5 mg/kg) or RTG (7.5 mg/kg) co-injected with XE991 (1 mg/kg) administration (i.p.) before 1% acetic acid (i.p.) injection in both wild type (WT) and transgenic (Tg) groups. Data are expressed as mean ± s.e.m (n = 8-13). RTG (7.5 mg/kg, i.p.) significantly reduced the number of writhes in WT mice, and co-injection of RTG (7.5 mg/kg, i.p.) with XE991 (1 mg/kg, i.p.) reversed the antinociceptive effect of RTG (*p < 0.05).

To further test the antinociceptive effect of retigabine, we performed i.p. injection of retigabine and tested its effect on visceral pain in both WT and Tg mice. Based on the similar responses to chemical stimulation with 1% AA in both WT and Tg groups (Figure 4B), we chose 1% AA to induce visceral pain and tested the effect of retigabine on the pain. Although no significant difference in onset time was found in Tg and WT groups, WT mice showed a tendency of longer onset latency when administered with retigabine, as compared with Tg mice (Figure 9A). In WT mice, retigabine decreased the total number of writhes induced by 1% AA by 51% (Figure 9B). In contrast, the antinociceptive effect of retigabine was less effective and showed a reduction of about 13% in Tg mice. The loss of the retigabine effect in Tg mice further confirmed the role of the KCNQ/M-channel in the forebrain, where channel function is important in modulation of visceral pain. The antinociceptive effect of retigabine was reversed by XE991 at a dose of 1 mg/kg, suggesting that the effect is specifically mediated by KCNQ channel activity.

## Discussion

### Central alteration of forebrain hyperexcitability in Tg mice

The aim of this study was to investigate whether altered activity of the brain can cause visceral pain hypersensitivity. Based on previously reported findings, we hypothesized that cortical alteration of membrane excitability may manifest as visceral pain hypersensitivity. However, to test this hypothesis requires a model with central alteration in a specific region of the brain to address the central effect of excitability on visceral pain hypersensitivity, which is experimentally challenging [4,23,24].

In this study, we adopted a transgenic approach by creating a mouse model that expresses a dominant-negative mutant of the voltage-gated KCNQ potassium channel under the control of the forebrain specific promoter αCaMKII. The αCaMKII promoter has been successfully used to achieve brain region specific expression of genes of interests [25-28]. To achieve central alteration of brain activity, we took advantage of voltage-gated Kv7/KCNQ channels whose mutations cause idiopathic generalized epilepsy of newborns [29,30]. Kv7/KCNQ channels are assembled as heteromultimers to encode the native M-current that is activated in the voltage range for action potential initiation, and is therefore an ideal molecular target for regulating the dynamics of the neuronal firing and excitability [13,14,31]. Suppression of M-current by muscarinic agonists (hence M-current) or Kv7/KCNQ blockers such as XE991 or linopirdine can cause an increase in intrinsic excitability [31]. By altering excitability using a transgenic approach, we have demonstrated that forebrain hyperexcitablity can induce visceral hyperalgesia, providing evidence for the critical role played by central alteration of brain-gut interaction in regulation of visceral pain.

### Co-localization of αCaMKII and KCNQ2 within the forebrain

αCaMKII is distributed throughout the forebrain, and is abundantly expressed in hippocampus, olfactory cortex, caudate-putamen, the septal nuclei, amygdala, thalamic nuclei and the substantia nigra [32,33]. In layers I, II and III of the cortex, αCaMKII shows a strong immunoreactivity [34]. KCNQ2 is expressed throughout the mouse brain, including the cortex, hippocampus, olfactory bulb, basal ganglia, septum and diagonal band, habenula and interpeduncular nucleus, thalamus reticular, lateral hypothalamic nucleus, midbrain, pon and cerebellum [35]. Thus, expression of KCNQ2 overlaps with the αCaMKII promoter in many areas of the forebrain such as the somatosensory cortex, insular cortex, prefrontal cortex (PFC), hippocampus and thalamus reticular. In this study, c-Fos expression in the somatosensory SII and insular cortex in response to acetic acid was higher in transgenenic mice than WT mice, illustrating that neuronal activity in these brain regions is higher in Tg mice than WT mice. Thus, neuronal hyperexcitability induced by suppression of KCNQ/M current in these forebrain regions likely manifests as visceral hyperalgesia in Tg mice.

αCaMKII expression is very low on postnatal day 4 and its expression does not reach adult level until day 16 [34], indicating that the early development of the transgenic mouse brain is most likely not affected by overexpression of the KCNQ2 pore mutation. This was further confirmed by our observation of normal hippocampal development in these transgenic mice. Therefore, the hyperalgesia observed with visceral pain stimulation in transgenic mice is most likely not due to altered neuronal development.

### Central sensitization mediates visceral hyperalgesia

Our transgenic mice showed activation of the c-Fos gene in the SII and insular cortex in response to visceral stimulation, but not in the SI cortex, which is consistent with clinical neuroimaging and animal studies that show visceral pain hypersensitivity and somatic sensation/pain are mediated by different cortical areas [36,37]. It is known that the cerebral visceral sensory/pain neuromatrix consists of both lateral and medial cortical somatosensory SI/SII areas, together with cingulate cortex/insula [9]. Somatic sensation can be localized precisely, whereas localization of visceral sensation is vague, suggesting there are different patterns of somatic and visceral input to the cerebral cortex [38]. fMRI studies have shown that visceral and somatic pain sensation is mediated by different cortical areas. Visceral stimulation results in activation of the insular cortex, anterior and posterior cingulate cortex, secondary somatosensory, sensory associations and prefrontal cortex, whereas somatic stimulation activation causes primary somatosensory cortex activation at a more superior level [36,38-40].

In this study, brain slice recording of hippocampal pyramidal neurons from Tg mice confirmed the presence of hyperexcitable properties with increased firing. Therefore, suppression of the Kv7/KCNQ channel in the forebrain increases neuronal excitability, and then most likely increases synaptic strength. Increased forebrain excitability and synaptic efficacy can produce functional changes and mediate central sensitization, which may help explain visceral hyperalgesia in KCNQ Tg mice.

## Conclusions

Our findings in this study show that visceral hyperalgesia is affected by alterations in cortical excitability, demonstrating that dysfunction of the brain-gut axis plays an important role in mediating visceral pain. Therefore, activation of KCNQ/M channel function has therapeutic potential for treatment of visceral pain which causes otherwise unexplained abdominal pain syndromes.

## Methods

### Generation of transgenic mice

The dominant-negative pore mutation of rat KCNQ2 was generated by mutating the residue 279-Gly to Ser (rQ2-G279S). rQ2-G279S cDNA was subcloned into a 265-plasmid with a forebrain-specific promoter of αCaMKII and an SV40 poly A. The αCaMKII promoter is specifically expressed in the forebrain and has been widely used for genetic manipulation of region- specific gene expression [25-28]. Transgenic mice of the B6D2F1/Crl strain were generated by pronuclear microinjection of linearized rQ2-G279S DNA in fertilized eggs. Transgenic mice were selected using genomic PCR with transgene-specific primers (upstream 5'-GCT AGA GGA TCT TTG TGT AAG GAA C-3', downstream 5'-GGA AAG TCC TTG GGG TCT TCT ACC-3'). Expression of the transgene coding for mutant rQ2-G279S in the forebrain was verified using real-time RT PCR and in situ hybridization.

### Animals

Female mice (both rQ2-G279S transgenic and wild type) of 8-12 weeks age were used in this study. In one experiment, ICR mice from the Laboratory Animal Center, Peking University Health Science Center were also used. Mice were housed in groups (two to five per cage) under a controlled temperature (23 ± 2°C) and humidity (50 ± 5%) environment with ad libitum access to food and water. Animals were maintained on a reverse 12 h/12 h light/dark cycle (lights on at 7:00 AM and off at 19:00 PM). The animal experimental protocols were approved by the Animal Use and Care Committee of Peking University and were consistent with the Ethical Guidelines of the International Association for the Study of Pain.

### Drug administration

The Kv7/KCNQ channel opener retigabine or the blocker XE991 (Tocris, UK) was dissolved in a mixture of tween-80 (Sigma, St. Louis, MO, USA) and saline (0.9% NaCl, autoclaved before use) at a ratio of 1:9 (v/v). For intracerebroventricular (i.c.v) injection, drugs were dissolved in DMSO and diluted with saline at a ratio of 1:1 (v/v). Drugs were diluted to desired concentrations one day before experiments and were stored at -20°C. Drug solutions were administered to mice intraperitoneally at a volume of 10 ml per kilogram body weight. Injection of drug solutions and behavior testing were conducted on the basis of a double-blind and randomized manner, in which one experimenter carried out drug injection and randomized division of mice, and another experimenter who was blinded to drug administration and mouse groups, conducted measurement of pain response.

### Patch clamp recordings of brain slices

Brain slices were transferred to a small volume (~0.5 ml) recording chamber that was mounted on a fixed-stage, upright microscope (Nikon, Japan) (IR-DIC) optics. The recording chamber was superfused with ACSF containing (mM): 125 NaCl, 25 NaHCO_3_, 2.5 KCl, 1.25 NaH_2_PO_4_, 1.5 MgCl_2_, 1.0 CaCl_2_, 16 glucose, 1.3 Na L-ascorbate, 0.6 Na-pyruvate and saturated with 95% O_2 _- 5% CO_2 _at a flow rate of ~2-3 ml/min. In some current-clamp experiments, 10 μm DNQX, 50 μm APV and 10 μm Gabazine were added to the extracellular medium to block spontaneous synaptic transmission. Patch electrodes were fabricated from filamented, thick-walled borosilicate glass pipettes and heat-polished to a resistance of 3~4 M when filled with an internal solution consisting of the following (mM): 130 K-gluconate, 10 HEPES, 0.6 EGTA, 5 KCl, 3 Na_2_ATP, 0.3 Na_3_GTP, 4 MgCl_2_, 10 Na_2_-phosphocreatine; pH adjusted to 7.25 with KOH. The final osmolarity of the solution was ~290 mOsm. Liquid junction potential (~7 mV) was not corrected. Recordings were made using a Multiclamp 700B amplifier (Molecular Devices, Union City, CA) operating in either voltage-clamp or current-clamp mode on the soma of CA1 pyramidal neurons. All recordings were acquired at 5 or 10 kHz with a Digidata 1440A interface (Molecular Devices) in conjunction with a PC, and filtered at 1 or 2 kHz, respectively, with a low-pass Bessel filter. Stimulus generation and data acquisition were performed using pClamp10 (Molecular Devices). The series resistance was 10-40 MΩ in whole-cell current clamp recording. Cells with a stable resting membrane potential and stable AP amplitudes (> 110 mV) were used, and all recordings were performed at room temperature (22°C ± 2).

### Locomotor activity

Locomotion was measured with the Animal Locomotor Video Analysis System (JLBehv-LAG-8, Shanghai Jiliang Software Technology Co. Ltd, China), which consists of eight identical black plexiglass chambers (30 ' 30 ' 65 cm) in light- and sound-controlled cubes. Each chamber was equipped with a video camera (winfast vc100) on the top. All cameras were connected to a PC computer for recording video files of movement of mice in chambers (n = 7 per genotype). The locomotor activity of each mouse was analyzed with the DigBehv analysis software (Shanghai Jiliang Software Technology Co. Ltd, China) and expressed as a total distance (cm) traveled in the period of 30 min [41].

### Behavioral responses and tests for visceral and cutaneous pain

#### Visceral pain induced by injection of capsaicin into the mouse colon

The chemical capsaicin was purchased from Sigma, and dissolved in 10% ethanol, 10% Tween 80 and 80% saline (according to 0.1% w/vol), and this solution of 10% ethanol, 10% Tween 80 and 80% saline was used as the vehicle. Mice were habituated to the glass observation chambers for 30 min before experiments. Petroleum jelly (Vaseline) was applied in the perianal area to avoid stimulation of somatic areas by contact with the irritant chemicals. 50 μl of chemical solution was administered by introducing a fine cannula with a rounded tip (external diameter 0.61 mm; 4 cm long) into the colon via the anus [42]. Spontaneous behavior was then observed directly over a period of 20 min. Postures defined as pain-related behavior were (i) licking of the abdomen, (ii) stretching the abdomen, (iii) squashing of the lower abdomen against the floor and (iv) abdominal retractions.

#### Visceral pain induced by i.p. injection of acetic acid or magnesium sulfate

After habituation in the glass observation chambers for 30 min, mice were given intraperitoneal (i.p.) injections (10 ml/kg) with 0.3%, 0.6%, or 1% acetic acid (n = 7-10 per genotype per concentration), and the number of lengthwise abdominal constrictions ("writhes") were counted over 15 min [43]. The concentration of magnesium sulfate was 12 mg/kg (n = 10 per genotype), and the number of writhes were counted over 10 min [23,44]. To test the antinociceptive effect of retigabine on visceral pain, mice received an intraperitoneal injection of vehicle or retigabine (7.5 mg/kg) or retigabine (7.5 mg/kg) co-injected with XE991 (1 mg/kg) at volume of 10 ml/kg, 20 min prior to 1% acetic acid injection (n = 8-13, per group). The vehicle used in this study was a mixture of tween-80 (Sigma, St. Louis, MO, USA) and saline (0.9% NaCl, autoclaved before use) in a ratio of 1:9 (v/v).

#### Tail-withdrawal test

While lightly restrained in a cloth holder, the distal half of mouse's tail was dipped into a bath of water thermostatically controlled at 47.0°C or 49.0°C (± 0.1°C) (n = 7-11, per genotype). The latency to respond to the heat stimulus by vigorous flexion of the tail was measured. Mice were tested for twice, and two latency determinations (separated by > 60 s) were measured and averaged [43].

#### Hot plate test

Mice were brought to testing room and allowed to acclimatize for 10 minutes before the test begins. Pain reflexes in response to a thermal stimulus were measured using a Hot Plate Analgesia Meter. The surface of the hot plate was heated to a constant temperature of 53°C (± 0.2°C). Mice were placed on the hot plate (25.4 cm × 25.4 cm), which is surrounded by a clear acrylic cage (19 cm tall, close top), and the Start/Stop button on the timer is activated. The latency to respond to hindpaw flick or lick was measured to the nearest 0.1 seconds by deactivating the timer when the response was observed. The mouse was immediately removed from the hot plate and returned to its home cage. If a mouse did not respond within 60 seconds, the test was terminated and the mouse was removed from the hot plate. Animals were tested one at a time and were not habituated to the apparatus prior to testing. Each animal was tested only once, because repeated testing can lead to systematic latency alterations [44-46].

#### Formalin test

After habituation to individual glass observation chambers, mice were given a subcutaneous injection of 5% formalin into the plantar right hindpaw (25 μl volume) and observed for 60 min after the formalin injection. The presence of right hindpaw licking/biting was recorded. The acute phase of formalin test was defined as 0-10 min after injection and the tonic phase as 10-60 min after injection. Data are presented as the time spent in each phase in which licking/biting was detected [43].

### Intracerebroventricular (i.c.v.) injection

Intracerebroventricular (i.c.v.) injections were made directly into the lateral ventricle (1 mm lateral and 0.4 mm caudal to the bregma at a depth of 2.5 mm) according to modification of a previously described method [47]. For i.c.v. injection, wild type mice were lightly anesthetized with ethylether. XE991, retigabine or vehicle (50% DMSO in saline) in 5 μl was injected directly into the lateral ventricle 15 min before 0.6% acetic acid (i.p.) injection. After behavioral evaluation, mice were sacrificed for examination of accuracy of the site of i.c.v injection. The dose of XE991 used was 30 nmol or 10 nmol, and the dose of retigabine was 49 nmol or 16 nmol based on our previous study [48].

### Tissue Preparations and Nissl straining

Adult mice were deeply anesthetized and transcardially perfused with normal saline followed by 4% paraformaldehyde. Brains were removed and post-fixed overnight at 4°C following submersion in 20% and 30% sucrose in 0.1 M phosphate buffer. Coronal sections were cut on a cryostat (Leica CM1900) into a series of 30 μm thick sections and mounted on glass slides for Nissl straining. For of c-Fos immunohistochemistry staining, 20 μm thick sections were cut and stored at -20°C in a solution containing 30% (v/v) ethylene glycol, 30% (v/v) glycerol, and 0.1 M sodium phosphate buffer. For Nissl straining, brain slides were stained with Nissl substance at 37°C for 1 hour and then rinsed with water, dehydrated in alcohol series and xylene, before coversliped with resinous mounting medium [22].

### c-Fos immunoreactivity

Free-floating sections were rinsed in 0.1 M PBS (pH7.4), incubated for 30 min in PBS containing 3% H_2_O_2 _at 37°C, and rinsed three times for 5 min each time in PBS. After 60 min incubation in 0.3% Triton X-100 in PBS containing 12.5% goat serum, these brain sections (n = 3-4) were incubated overnight at 4°C with c-Fos primary antibody (1:1000; Santa Cruz Biotechnology). Sections were rinsed for 5 min in PBS (three times) and incubated for 60 min with secondary antibody (Zhongshan Golden Bridge Biotechnology company, Beijing, China). After rinsing three times in PBS, the reaction was detected using 3, 3'-diaminobenzidine (DAB). Analysis of c-Fos staining was carried out by an observer who was blinded to the genotype and drug treatment of the mice. Cells containing a nuclear brown-black reaction product were considered to be c-Fos-positive. All c-Fos-positive cells that were distinguishable from background staining were counted in each region of interest within a defined area (0.01 mm^2^) [49].

### In situ hybridization

PCR primers used for amplifying the transgene vector SV40 probe were as follows: upstream primer 5' GAGATGTGGCTTGGGCAG 3' and downstream primer 5' CATTCATCAGTTCCATAGGTT 3'; and the PCR product was 248 bp in size. Using the Gel DNA Extraction kit (Tiangen Biotech, Beijing, China), the resulting PCR product was extracted from the gel and subcloned into the pSPT18 vector with T4 ligase (Takara). The identity of the plasmid was confirmed by DNA sequencing. The plasmid was linearized with HindIII or EcoRI and used for RNA transcription with T7 or Sp6 RNA polymerase to generate antisense or sense probes, respectively, in the presence of digoxigenin-labeled rUTP (Roche Diagnostics). Deparaffinized and dehydrated paraffin-embedded tissue sections (5 μm) were incubated in 0.1 M HCl for 10 min, and treated with Proteinase K (20 μg/ml in TBS which contains 2 mM CaCl2) for 20 min at 37°C. The slides were cooled to room temperature, washed with TBS (150 mM NaCl, 50 mM Tris, pH7.5), fixed in 4% paraformaldehyde for 10 min, and hybridized overnight at 47°C with the SV40 cRNA probe. After hybridization, sections were washed in 2xSSC plus 50% formamide for 30 min and 2xSSC twice for 15 min (37°C). The samples were incubated with antidigoxigenin antibody conjugated with alkaline phosphatase (dilution 1:500; Roche) for 1h. 5-Bromo-4-chloro-3-indolyl phosphate and nitro-blue-tetrazolium (Roche) were used for the color reaction [50].

### Real time RT-PCR

60 μg of RNA extracted from mouse brain were digested with 6 μl Turbo DNase for 1 hour. cDNA was synthesized using oligo dT and M-MLV reverse transcriptase according to the manufacturer's instructions (Invitrogen). The PCR amplification mixtures (20 μl) contained 1 μl template cDNA, 10 μl 2 × SYBR^® ^Premix Ex TaqTM (Takara) containing SYBR Green and 5 μM forward and reverse primers. The sequences of these primers (for β-actin, upstream 5'-TGT TAC CAA CTG GGA CGA C-3', downstream 5'-GGT GTT GAA GGT CTC AAA CAT-3'; for rQ2-G279S, upstream 5'-CTC TGC CAC TGG TGA AGG-3', downstream 5'-TGT AGG TAG TTT GTC CAA TTA TGT C-3', for genomic DNA primers, upstream 5'-CCC ATA CAC CTC CTC TGA A-3', downstream 5'-CTG AAG TGT CTA CCC TTA CGG-3') were designed using Oligo software. The product of the rQ2-G279S primers used to amplify the KCNQ2 mutant gene within SV40 poly A was 160 bp. The genomic DNA primers located in the 265-plasmid after SV40 poly A were used to detect any contamination of transgenic genomic DNA with product length of 148 bp. β-actin was used as an internal reference and the product length was 163 bp. Amplification was carried out using the ABI PRISM 7500™ with cycling conditions as follows: there was an initial denaturation step at 95°C for 10 s, followed by 40 cycles at 95°C for 5 s and 60°C for 34 s with fluorescent detection at 60°C. Melting curve analysis was performed from 60°C to 95°C in 1°C step. Results were analyzed using the 2^-ΔΔCt ^method to compare expression of genes of interest with that of β-actin [51].

### Data analysis

Statistical analysis was performed with GraphPad Prism (the 5th edition) and SPSS 13.0 for Windows. All data are presented as mean ± s.e.m. Statistical significance between multiple groups was examined by the Student's *t *test or one-way or two-way ANOVA with an appropriate post hoc test. A value of *p *< 0.05 was considered to be statistically significant.

## Abbreviations

AA: acetic acid; ACC: anterior cingulated cortex; ANOVA: analysis of variance; CNS: central nervous system; DMSO: dimethyl sulfoxide; DRG: dorsal root ganglion; FGIDs: functional gastrointestinal disorders; fMRI: functional Magnetic Resonance Imaging; IC: insular cortex; i.c.v.: intracerebroventricular; i.p.: intraperitoneal; Mor: Morphine; PET: positron emission tomography; PFC: prefrontal cortex; rQ2-G279S: dominant-negative pore mutation of rat KCNQ2 in which a glycine residue was mutated to serine; RTG: retigabine; S.E.M.: standard error of mean; SPECT: single photon emission computed tomography; Tg: transgenic; WT: wild type.

## Competing interests

The authors declare that they have no competing interests.

## Authors' contributions

YB and KWW designed the experiments. YB carried out behavioral assays and histological experiments and drafted the manuscript. HC carried out genome typing and participated in behavioral assays. JS generated the transgenic mice and participated in double blind behavioral experiments. XC and XB carried out electrophysiological experiments. KWW finished the final draft of the manuscript. All authors read and approved the final manuscript.
